# A scalable convolutional neural network approach to fluid flow prediction in complex environments

**DOI:** 10.1038/s41598-024-73529-y

**Published:** 2024-10-04

**Authors:** Pratip Rana, Timothy M. Weigand, Kevin R. Pilkiewicz, Michael L. Mayo

**Affiliations:** 1 Bennett Aerospace, Vicksburg, 39180 USA; 2 Oak Ridge Institute for Science and Education, Oak Ridge, 37830 USA; 3 U.S. Army Engineer Research and Development Center, Environmental Laboratory, Vicksburg, 39180 USA

**Keywords:** Computational fluid dynamics, Deep learning, Convolutional neural networks, Fluid dynamics, Computer science

## Abstract

We evaluate the capability of convolutional neural networks (CNNs) to predict a velocity field as it relates to fluid flow around various arrangements of obstacles within a two-dimensional, rectangular channel. We base our network architecture on a gated residual U-Net template and train it on velocity fields generated from computational fluid dynamics (CFD) simulations. We then assess the extent to which our model can accurately and efficiently predict steady flows in terms of velocity fields associated with inlet speeds and obstacle configurations not included in our training set. Real-world applications often require fluid-flow predictions in larger and more complex domains that contain more obstacles than used in model training. To address this problem, we propose a method that decomposes a domain into subdomains for which our model can individually and accurately predict the fluid flow, after which we apply smoothness and continuity constraints to reconstruct velocity fields across the whole of the original domain. This piecewise, semicontinuous approach is computationally more efficient than the alternative, which involves generation of CFD datasets required to retrain the model on larger and more spatially complex domains. We introduce a local orientational vector field entropy (LOVE) metric, which quantifies a decorrelation scale for velocity fields in geometric domains with one or more obstacles, and use it to devise a strategy for decomposing complex domains into weakly interacting subsets suitable for application of our modeling approach. We end with an assessment of error propagation across modeled domains of increasing size.

## Introduction

Despite significant advancements in computational hardware and numerical methods, accurate, real-time computational fluid dynamics (CFD) simulations remain unattainable. An increasing demand for real-time simulation has led the research community to develop less complex approximation methods in favor of advances to optimization and efficiency of numerical integration schemes. A need for real-time CFD simulation exists across a broad range of industries, such as in emergency management, wherein flow simulations aid in building evacuation plans in the event of fire^1^. Other applications range from the analysis, control, and optimization of online industrial processes^2^ to visualization in computer games^3^. Existing approaches for generating real-time CFD simulations involve either approximating the fluid-flow equations, which trades predictive accuracy for an increase in computational speed (e.g., fluid visualizations in video games), or the pre-generation of CFD simulations, which can be used to create reduced-order or empirical models for rapid evaluation of the velocity (flow) fields, or the CFD results can be coarsely discretized and then directly interpolated for an ad hoc evaluation of the fluid state^4^.

Machine learning models offer another means to assimilate pre-generated CFD results to produce fluid predictions that are accurate, yet available, in an accelerated or near real-time capacity. Machine learning approaches designed to decrease the computational time of CFD simulations while maintaining accuracy can be split into methods that aim to increase the rate of convergence of numerical simulators and methods that produce output variables solely from input data^5,6^. Methods from the former category have the benefit of explicitly satisfying convergence constraints of the underlying mathematical model but may still require significant computational time. While physics-informed loss functions can be utilized in methods from the latter category, these models cannot yet be used to meet a user-specified convergence criteria^7^. The latter approach, however, shifts the computational burden toward parameter identification (i.e., training), wherein model evaluation is nearly instantaneous and can be used in time sensitive applications, which is the focus of the present work.

Developing and training a machine learning model to reproduce flow characteristics in complex physical domains is challenging in itself, but to generalize this capability to new interface geometries and obstacle configurations creates an additional challenge that machine learning methods might be poised to address. For example, deep learning architectures, such as U-net^8^, have been shown to learn from CFD data efficiently^9,10^. Improvements to the original U-Net have been made by modifying the architecture, such as by including gated residual blocks^11^, connecting multiple U-net architectures^12–14^, and adding long short term memory layers^15^ to decrease training times and/or improve solution accuracy.

A major drawback for the U-net architecture, and convolutional neural networks (CNNs) in general, is that solutions are restricted to a Cartesian grid. Furthermore, the grid resolution may not be changed without retraining the model. Approaches to relax this restriction have been successful, e.g., for unstructured grids, although in this case the number of vertices where a solution is provided remains fixed, but vertices can be placed in alternative locations across the domain^16^. A model with a fixed grid size (or a set number of vertices) is ultimately unable to simulate larger, more complex domains without retraining, which constrains its utility. Model training remains a computationally intensive endeavor that requires a vast amount of representative data to establish an acceptable level of predictive accuracy. It is therefore advantageous to develop machine learning models that avoid the need for frequent retraining by being accurate across a broad range of initial conditions and domain geometries.

One approach to meet this goal is to reduce the informational content of dynamic velocity fields by trading the complexity of short-term density fluctuations for the long-term predictability of steady-state flows, as exemplified for both singular obstacles^9^ and porous media systems^17^. These two studies illustrate how CNNs can be used to reconstruct steady state velocity fields within spatially heterogeneous domains. However, if the domain of interest were expanded (e.g., by altering the grid size), then each of these models would need significant retraining in addition to a revalidation against higher-resolution source data. Is it possible, then, to build upon these steady-state modeling successes and develop a methodology for the prediction of fluid flows in arbitrarily large domains?

Here we propose a domain decomposition approach that leverages a characteristic structural scale for which velocity field predictions are effectively decoupled from those of the other subdomains. Velocity field predictions developed for these individual subdomains can then be recombined into a patchwork that collectively models a continuous velocity field that spans the domain of interest. A key feature of this approach is that velocity fields can be learned using simpler model architectures than otherwise expected when models are trained to velocity field states across the entirety of the full domain. This advancement makes it possible to predict velocity fields across domains of arbitrary size using just a single model with a fixed number of trained parameters (albeit with error that scales with system size), whereas intuition suggests the number of model parameters (i.e., number of network layers, larger networks) should increase with system size, because a larger domain encodes more velocity states. One caveat is that larger models may be more prone to experience issues such as over-fitting, or the vanishing and exploding gradient problem.

This paper is organized as follows. The “Materials and methods” section describes production of simulated CFD datasets and neural network model architectures. The “Results” section describes our modeling approach, namely how velocity fields can be predicted for a single domain, and then how these individual regions can be combined to predict the velocity fields of arbitrarily large domains. Finally, the “Discussion” section reflects on our lessons learned and concludes the discussion.

## Materials and methods

### CFD dataset

Two-dimensional velocity fields–referred to herein as “flow” fields–were estimated from CFD simulations performed using the open-source, finite volume simulation software OpenFoam (version 1912)^18^. OpenFoam was used to generate all domain geometries and their tessellations throughout this work. In general, our simulations involve laminar and transitional fluid flows around convex obstacles, and avoid fully turbulent flow regimes.

#### Obstacle characteristics and computational generation of domains

Domain decomposition methods partition a spatially complex or heterogeneous domain into a set of smaller, more elementary elements that collectively approximate the original domain. For some domains, the choice of such elements is straightforward. Urban domains, for example, represent a complex arrangement of box-like structures that vary in position, orientation, and shape. A traditional approach describes the urban structural environment in terms of a regular array of parallelpipeds^19^ to account for differential exchange of energy at boundaries with atmospheric flows^20^. At much smaller scales, granular domains such as hydrogels^21^ and filtration systems^22^ can be described in terms of elementary spheres or discs. Due to their geometrical simplicity and potential to generalize, we focus here on modeling disc and square obstacles to fluid flow throughout our domains of consideration.

We trained/tested our model on circular or rectangular obstacles in various orientations. The position, orientation, and characteristic lengths (diameter/side length) of each circular or rectangular obstacle was chosen by sampling an appropriate uniform distribution. All relevant lengths were scaled relative to the domain side length. For domains enclosing a circular object, radii were restricted to the interval [0.1, 0.3]; for rectangular obstacles, side lengths were selected from the interval [0.1, 0.6]. The center of each obstacle was selected to ensure that all obstacles were fully located within the domain, and that their edges were no closer than a distance of 0.05 to any domain boundary.

Within OpenFoam, the mesh-generating tool snappyHexMesh was used to transform the structured background mesh into an unstructured mesh. A grid resolution study was performed to determine the necessary grid size for the background mesh and the refinement of obstacle surfaces. Using a dense grid solution and the $L_2$ error norm of the magnitude of the velocity, a value of $10^{-6}$ was selected as the cutoff for a resolved computational grid. At this value, a background mesh of 50 by 50 cells was used with a surface refinement level of two. For domains with multiple obstacles, the background mesh was scaled such that each subdomain had the correct grid-independent resolution.

#### Parameters of flow simulation

To simulate the fluid flow around obstacles, the time-dependent, incompressible, Navier-Stokes equations were solved numerically using OpenFoam’s icoFoam solver with “no-slip” boundary conditions applied to obstacle boundaries, in which fluid flow vanishes at surface boundaries. A fixed flow velocity oriented parallel with two of the rectangular domain boundaries was applied to one of the four domain boundaries, referred to here as the “inlet.” Zero velocity gradient and pressure conditions were applied to the boundary on the opposite side as the inlet (termed the “outlet”), and symmetry conditions were applied to the two remaining boundaries transverse to the direction of mean inlet flow. For each flow simulation for a given domain, multiple inlet speeds were used, and the resulting Reynolds number ranged from approximately 2 to 350. To pass these simulation data to a deep-learning model, described below, the computational domain was discretized post-simulation into a 128x128 structured grid and the flow velocity field subsampled to this resolution. To account for dynamically oscillating and spatially periodic flow patterns that can develop, we time-averaged velocities at each node of the domain grid.

### Neural network model architecture

**Figure 1:**
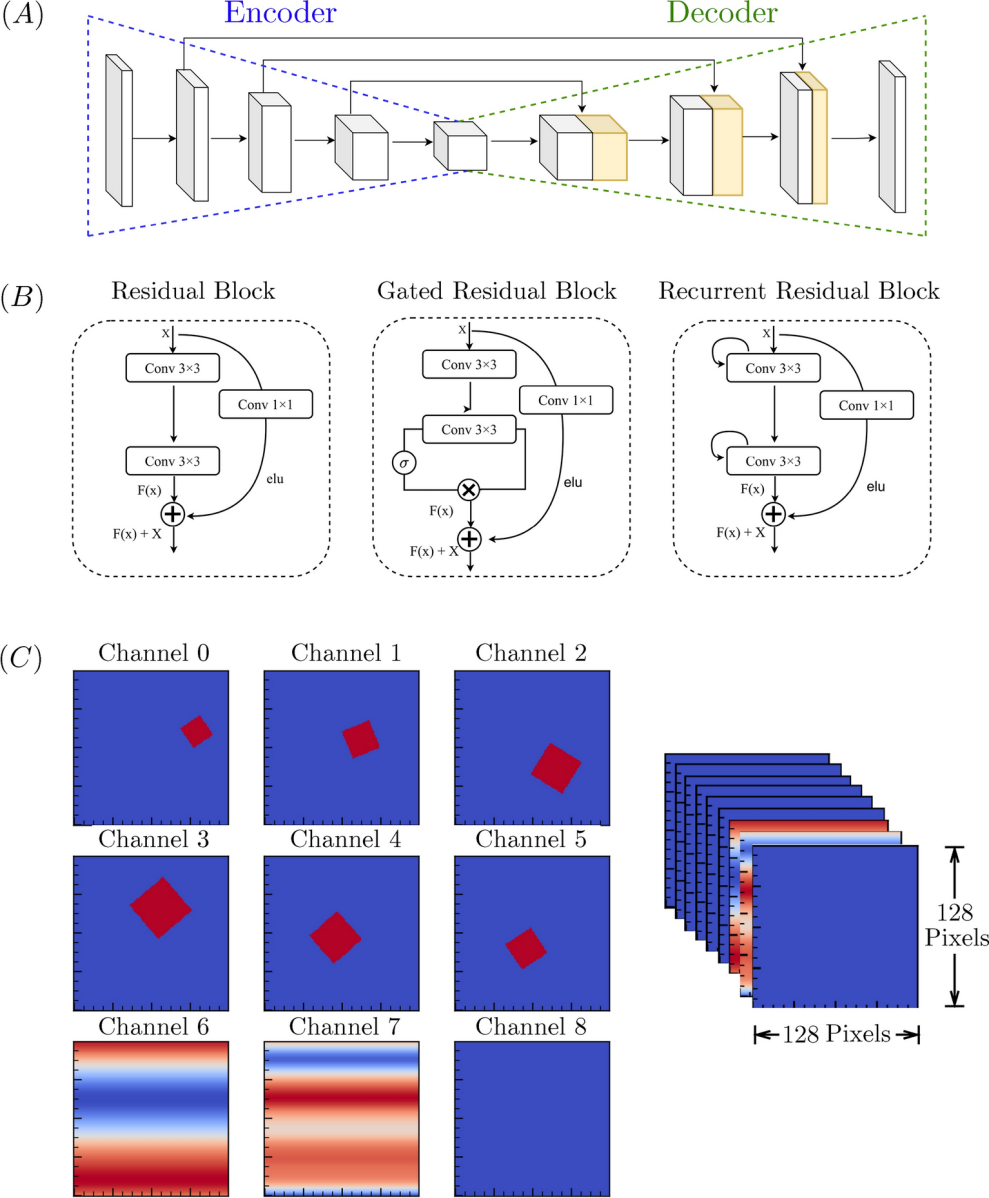
Architectures of the deep learning model. (**A**) Architecture of Gated Residual U-net, which consists of one encoder, one decoder, and multiple skip connector units. The encoder incorporates a convolution with stride 2 down-scaling, and the decoder employs a deconvolution layer with stride 2 for up-scaling. (**B**) (Left) Building block of Residual U-net, in which the 1D convolution enables residual learning. (Middle) Building block of Gated Residual U-net. (Right) Building block of Recurrent Residual U-net. (**C**) Nine different feature channels of input dataset. Inlet velocity is directed from left to right.

As illustrated in Fig. 1A, we used a gated residual U-net as the primary deep learning (DL) architecture for predicting fluid velocity fields, which is modified from the work of Hennigh^11^. This architecture consists of one encoding part, one decoding part, and several skip-connection layers similar to U-net. The encoder maps a high-dimensional tensor to a lower-dimensional latent representation. In contrast, the decoder reconstructs an approximate tensor from the lower dimensional latent representation. The skip connection layers directly connect the encoder layer to the decoder layer, which helps to adapt to information lost by the encoding process. Skip connections also help to retain high-level and low-level features to reconstruct the output. The encoder consists of multiple down-scaling gated residual blocks, while the decoder consists of multiple up-scaling gated residual blocks. For down-scaling, we used a convolution of stride 2; for up-scaling, we used a deconvolution (also known as transpose-convolution) layer with stride 2. All CNN kernels, i.e., the size of the convolution filters, were chosen as 3x3. We also applied “SAME” padding, so the convolution layers’ output had the same spatial dimension as the input. The initial number of channels was set to 8, after a performance analysis which varied these channels from 8 to 32. Like the original U-net architecture, the number of channels was doubled for every down-scaling operation and halved for each upscaling operation. After the decoding step, we performed a final convolution to reduce the output size to two channels representing each of the two orthogonal components of the velocity field.

We used a gated residual unit as the basic building block of the encoder and decoder units, as illustrated in Fig. 1B (Middle). This unit enables residual learning to build a deeper architecture without overfitting the model^23^. For the activation function of the gated residual unit, we chose the Exponential Linear Unit (ELU)^24^, which is defined by the following expression:

1 $$\begin{aligned} \text {ELU}(x) = {\left\{ \begin{array}{ll} x & \text { if }x>0\\ e^x-1 & \text {if} x \le 0 \end{array}\right. } \end{aligned}$$

However, alternative activation functions, such as the Rectified Linear Unit (ReLU), can be used here. We also used a sigmoid activation after each gating transformation and a hyperbolic tangent activation function after the last convolution layer. Finally, we used the standard dropout^25^ and $L_2$ regularization^26^ methodologies to prevent overfitting, wherein model predictions may become overly sensitive to minor variations in training data and thus reduce performance.

In Fig. 1B we also contrast our chosen gated residual unit with two other common U-net building blocks. The standard residual U-net block (Left) implements residual connections inside each encoder and decoder block. A recurrent residual U-net^27^, on the other hand, leverages a recurrent residual convolution block in each encoder/decoder unit (Right) that can accumulate features inside the model, which may improve predictive accuracy.

Our model input consists of nine different feature channels as shown in Fig. 1C. The first channel encodes a binary representation of each object and the boundary conditions of the domain. The following five channels consist of the binary representation of the obstacle(s) outside of the prediction domain in the left, right, front, left-front, and right-front directions, labeled with respect to the inlet flow direction. These channels enable the model to learn velocity field components near an obstacle, even in the presence of other nearby obstacles. We have ignored obstacle location information from the domain’s back, back-left, and back-right regions because the inlet velocity can encode that information into the model due to a minimal presence of counter-current flow in the areas immediately preceding an obstacle. The final three channels are related to components of the domain inlet velocity. Specifically, these three channels are the normalized $\hat{x}$ and $\hat{y}$ components of the inlet velocity, in addition to a normalized absolute inlet velocity.

### Model training and hyperparameter selection

Unless otherwise specified, we partitioned our datasets into three disjoint subsets to support model training and validation: a) training data ($64\%$), b) validation data ($16\%$), and c) test data ($20\%$). The model was trained via back-propagation using the Adam optimizer with a learning rate of $10^{-3}$ and optimal hyperparameters were selected based on the performance of model validation. Several hyperparameters have been learned to optimize the model performance, such as kernel size, the number of training epochs, the number of upscaling and downscaling layers, dropout rate, and regularization coefficients. Model performance was then evaluated on the test data partition.

In our model, we use the standard mean square error (MSE) loss function, which is the preferred choice for regression tasks in a deep learning approach. It is defined as

2 $$\begin{aligned} \text {MSE} = \frac{1}{N} \sum _{i=1}^N(v_{i} - \hat{v}_{i})^2, \end{aligned}$$

wherein $v_{i}$ and $\hat{v}_{i}$ are, respectively, the ground truth and predicted velocity at the $i^{th}$ grid point, and *N* is the total domain-specific number of grid points.

## Results

### A local contiguous model for flow prediction in a single domain

**Figure 2:**
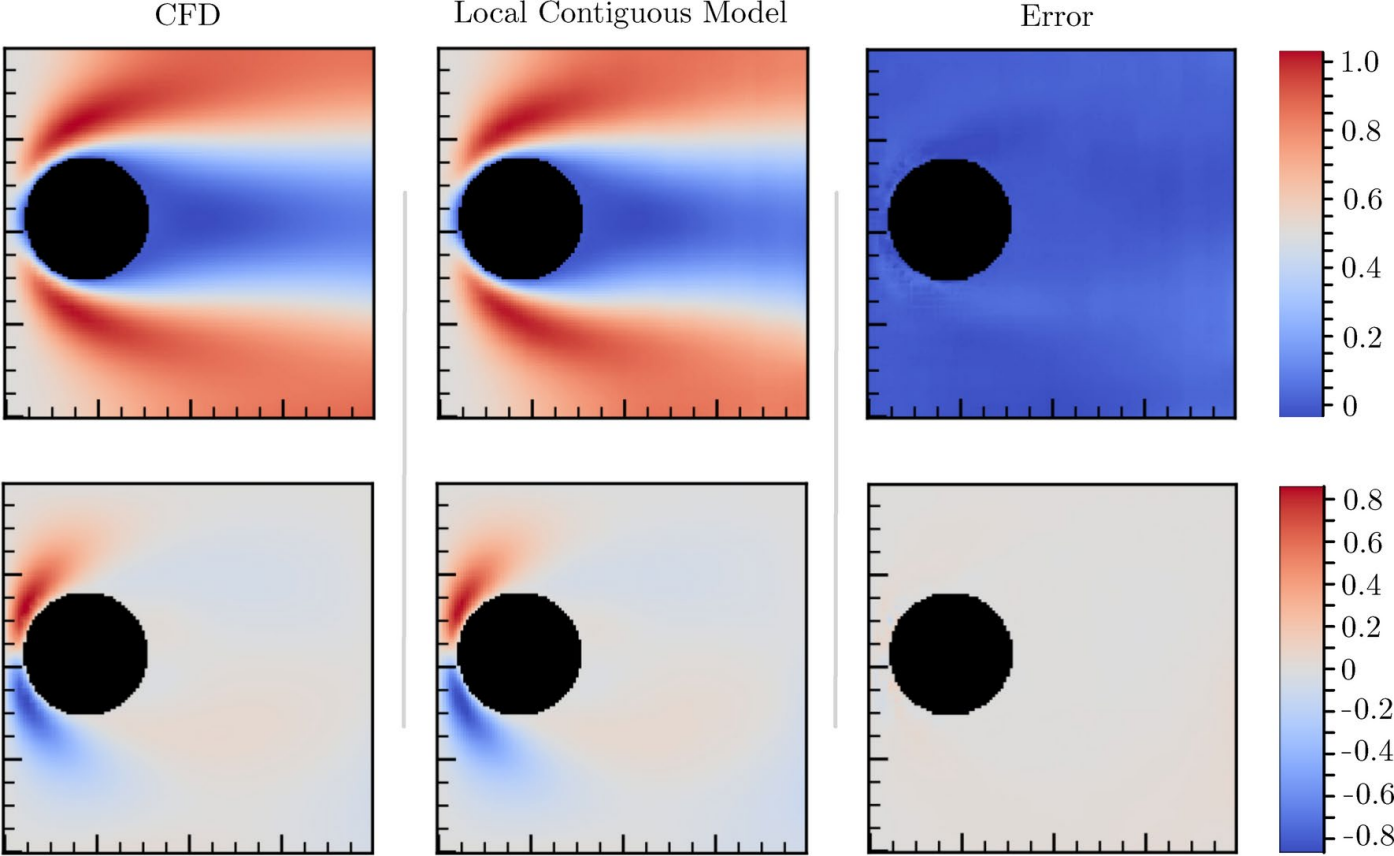
Visualization of model performance. Columns, from left to right: CFD simulation of the single domain, deep learning model prediction, and prediction error on a 128 $\times$ 128 resolution domain. The first and second rows are, respectively, the $\hat{x}$ and $\hat{y}$ flow velocity components. Error reflects the absolute difference between CFD and local contiguous modeling approaches. Error bars are scaled by a factor of $10^3$ for visual clarity. Inlet velocity is directed from left to right.

We surveyed the performance of several algorithms trained with CFD data containing just a single domain and one obstacle, and refer to them as “local contiguous” models. In general, we find that a gated residual U-net architecture fits the CFD generated training data effectively, returning relatively low MSE values for test data of different sizes and complexity. An illustration comparing ground-truth CFD flow, predicted flow, and absolute flow difference for a Reynolds number of 292 is shown in Fig. 2. We noticed that MSE values increase with the inlet velocity, because larger inlet velocities shift flow patterns toward the transition to the turbulent domain, which is fundamentally complex and therefore harder to predict. Similarly, MSE values are higher near obstacle boundaries and lower elsewhere.

Performance benchmarks for several deep-learning architectures in relation to flow prediction for a fixed inlet velocity were conducted for (a) U-net, (b) Residual U-net, (c) Gated Residual U-net, (d) Nested U-net^28^, and (e) recurrent Residual U-net^27^ algorithms. MSE values for these architectures, calculated on test data with a domain resolution of 128 × 128, are provided in Table 1. The main takeaway is that including a residual layer and gating improved model performance over the standard U-net, at the cost of only $\sim 36 \%$ increase in the number of parameters. The $\sim 5.8 \%$ increase in accuracy achieved by the Recurrent Residual U-net was deemed not worth the the reduced stability and $\sim 30$ min longer training time.

**Table 1:** Validation loss and approximate time to train the model for different benchmarked models.

Architecture	Parameters	MSE	Time (min)
U-net	1,081,914	0.0471	90
Residual U-net	1,475,946	0.0350	200
Gated residual U-net	2,016,858	0.0324	240
Nested U-net	566,586	4.2372	330
Recurrent Residual U-net	3,306,154	0.0305	270

Gated residual U-net and Recurrent U-net achieved the lowest mean-square error (MSE) on test data.

**Figure 3:**
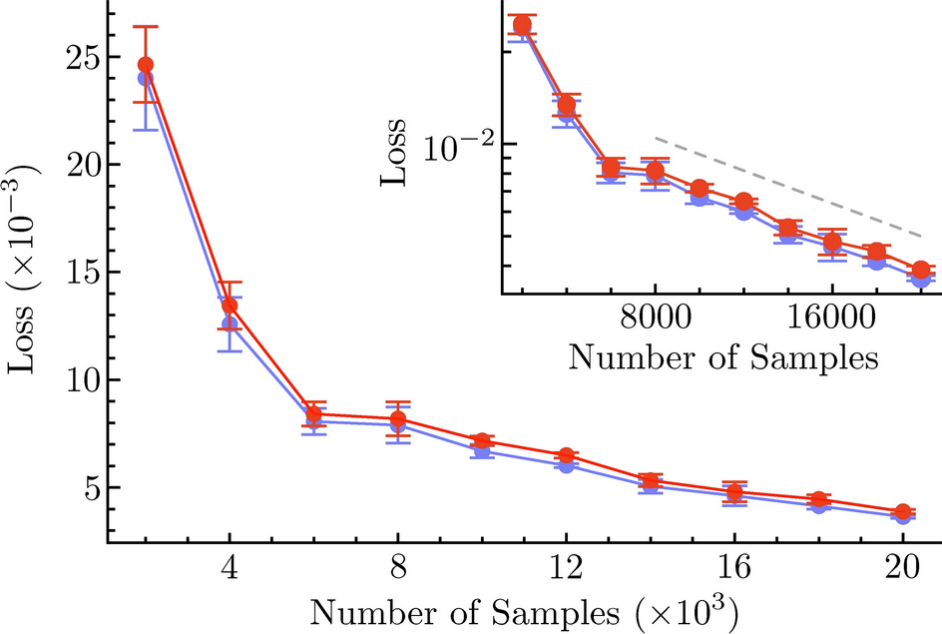
Five-fold cross-validation of the single domain model. Loss (error) in relation to the validation data is shown in red, whereas loss associated with the test datasets is shown in blue. Error bars represent standard deviations of loss for each finite set of CFD data chosen. (Inset) Five-fold cross-validation of the single domain model where the y-axis (loss) is in log scale. Straight (dotted) line in the logarithm scale indicates exponential decay, provided for reference.

We performed an extensive five-fold cross-validation of the deep learning model, illustrated in Fig. 3. The *k*-fold cross-validation method is reliable for measuring model performance when data availability is limited. First, we chose between 2000 and 20,000 domains with different inlet velocities and obstacle types from our library of CFD simulations. We then partitioned these data into $k=5$ disjoint sets via uniform random sampling, and used four of them ($k-1$) for model training, and used the remaining set for model validation. Figure 3 demonstrates a low standard deviation in the loss, which confirms that the gated, recurrent U-net architecture achieved stable performance with respect to flow prediction. The loss initially decreases very sharply as the number of sample domains included in the cross-validation methodology is increased; after about 6000 samples, however, the loss begins to decrease at a much slower rate that is well fit by an exponential decay (see the inset to Fig. 3). The degree of similarity in model performance across test and validation datasets indicates an absence of overfitting.

### Scaling to arbitrarily large domains

Large domain flow simulation is challenging because higher grid complexity generally requires more computations to accurately solve the governing equations. We intend to side-step this problem by leveraging our single-domain, locally contiguous model to describe flow around an arbitrary number of additional obstacles. In larger domains, flow estimation over a greater number of points tends to decrease single-domain model performance. For example, a five-layer architecture that predicts flow within a domain with $128 \times 256$ pixel resolution is required to achieve near-identical performance to that of a four-layer architecture predicting flow within a $128 \times 128$ pixel domain. While this suggests that increasing the number of hidden layers in the model could potentially address this issue, generalizing this approach to arbitrarily large domains will run a high risk of producing vanishing or exploding gradients. Another challenge is that large resolution flow-field domains require an equally large training dataset for accurate flow prediction. Generating these data using computational fluid dynamics simulations is not practical due to the excessive simulation time.

#### Flow prediction at arbitrary scales via naive patching: the contiguous model

To extrapolate our Local Contiguous model to an arbitrarily large planar domains with multiple obstacles, we first decompose each such domain into a set of non-overlapping subdomains of equal dimension. We restrict our consideration to domains that can be decomposed into subdomains that extend both horizontally and vertically, with one obstacle present in each subdomain of equal size. For example, a $384 \times 384$ pixel domain is first decomposed into 9 subdomains, $S_{ij}$, each of size $128 \times 128$, with horizontal and vertical indices (relative to “flow”) labeled $i, j \in \{ 1,2,3 \}$, respectively. Similar domain decomposition-based technique has been used previously to generalize fluid flow prediction from a deep-learning model^29^. Next, we use the Local Contiguous model to predict the velocity fields for the subdomains ($S_{1j}$) bordering on the domain inlet, as described above. For each subdomain $S_{1j}$, the predicted outflow velocity field then gets used as the inflow boundary data for the adjacent subdomain, $S_{2j}$.

Iterating this process, we gradually predict the flow fields for all subdomains. We then stitch these predicted subdomain flow fields back together into a patchwork that approximates the velocity field of the original large domain. We refer to this patchwork approach to domain scaling as the Contiguous model, to distinguish it from our Local Contiguous approach for predicting flows within just a single domain.

**Figure 4:**
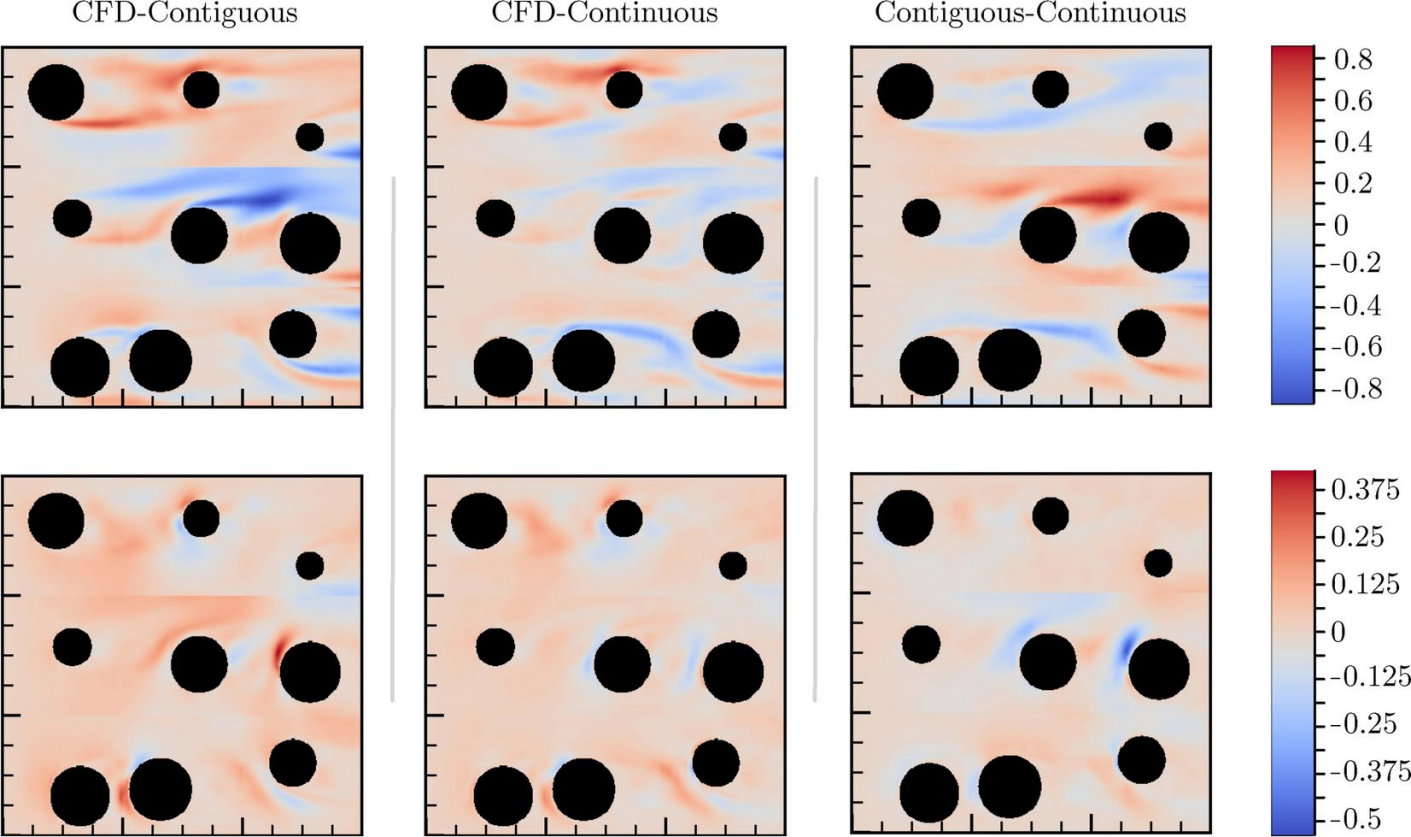
Each column illustrates a pairwise error calculation between model predictions. Left, middle, and right columns show, respectively, errors between CFD and Contiguous model, CFD and Continuous model, and Contiguous model and Continuous model. The top row denotes error in $v_x$ component of the velocity field, whereas the bottom row illustrates error in the $v_y$ component. Error bars are scaled by a factor of $10^3$ for visual clarity. Inlet direction is from left to right.

Differences between CFD (ground-truth) flow fields and predicted flows for just one configuration are shown in Fig. 4 (left column). This system is initialized with a constant flow at $x=0$ in the $\hat{x}$ direction. The top row of the domain, $S_{1j}$, has the lowest error, which increases gradually with distance away from this source (i.e., increasing *x*). Note that velocity field predictions at horizontal boundaries of adjacent domains are neither continuous nor smooth. We address this problem by feeding flow predictions for the entirety of the reconstructed domain into yet another deep-learning model, described in the next section.

#### Smoothing discontinuous flows across adjacent subdomain boundaries: the continuous model

To reduce the errors introduced by the naive patching method of the Contiguous model, explained above, we developed another deep learning model, which we refer to as the Continuous model. Our Continuous model attempts to enforce smoothness and continuity conditions in velocity fields predicted by the Contiguous model at adjacent subdomain boundaries. This Continuous model uses a gated, residual U-net architecture; however, inputs of this model consist of only three feature channels. The first channel is the binary representation of the joined subdomains. The other two channels are the $\hat{x}$ and $\hat{y}$ components of the velocity fields predicted from the Contiguous model. We split training datasets into two subsets in an effort to improve generalization and reduce the risk of model overfitting. One subset was used to train the Contiguous model, whereas the other was used to train the Continuous model.

The application of the Continuous model considerably improves flow predictions over those from the stand-alone Contiguous model. The second column of Fig. 4 illustrates the difference in velocity field components between the CFD and Continuous model. The third column of Fig. 4 contrasts flows between the Contiguous and Continuous models. It’s clear from this example that the Continuous model lowers overall predictive error by increasing or decreasing the values of the velocity field components predicted by the Contiguous model. This is especially notable in the apparent improvement of continuity and smoothness at the subdomain boundaries.

### Optimal subdomain partitioning and error propagation across reconstructed domains

When a uniform inlet flow passes around an obstacle, information about the size and shape of the obstacle is encoded as a disturbance to the flow velocity field. As this disturbance flows downstream from the obstacle, the information about its source is lost to dissipative forces in the fluid. This suggests that the ideal way to minimize subdomain patching errors in our deep learning model is to choose subdomain boundaries that are far enough downfield from any upstream obstacles that the flow field approximately returns to its unperturbed inlet profile. Any more complicated velocity profile will have to be predicted imperfectly by the deep learning algorithm, and even small errors at the first subdomain boundary will eventually compound into large errors as the flow field is predicted across succeeding subdomains of a large region of interest.

We will define the decorrelation length scale as the minimal distance one must travel downstream from an obstacle in order to see the flow return to its unperturbed state. Focusing our attention on channel and uniform inlet flow, as discussed in the “Parameters of flow simulation” subsection and used throughout this paper, it is clear that the decorrelation length will depend upon the size and shape of the obstacle as well as the Reynolds number of the fluid. For a given obstacle and inlet flow speed, we can quantify the corresponding decorrelation length by combining CFD calculations of the true flow past the obstacle with ideas from information theory.

**Figure 5:**
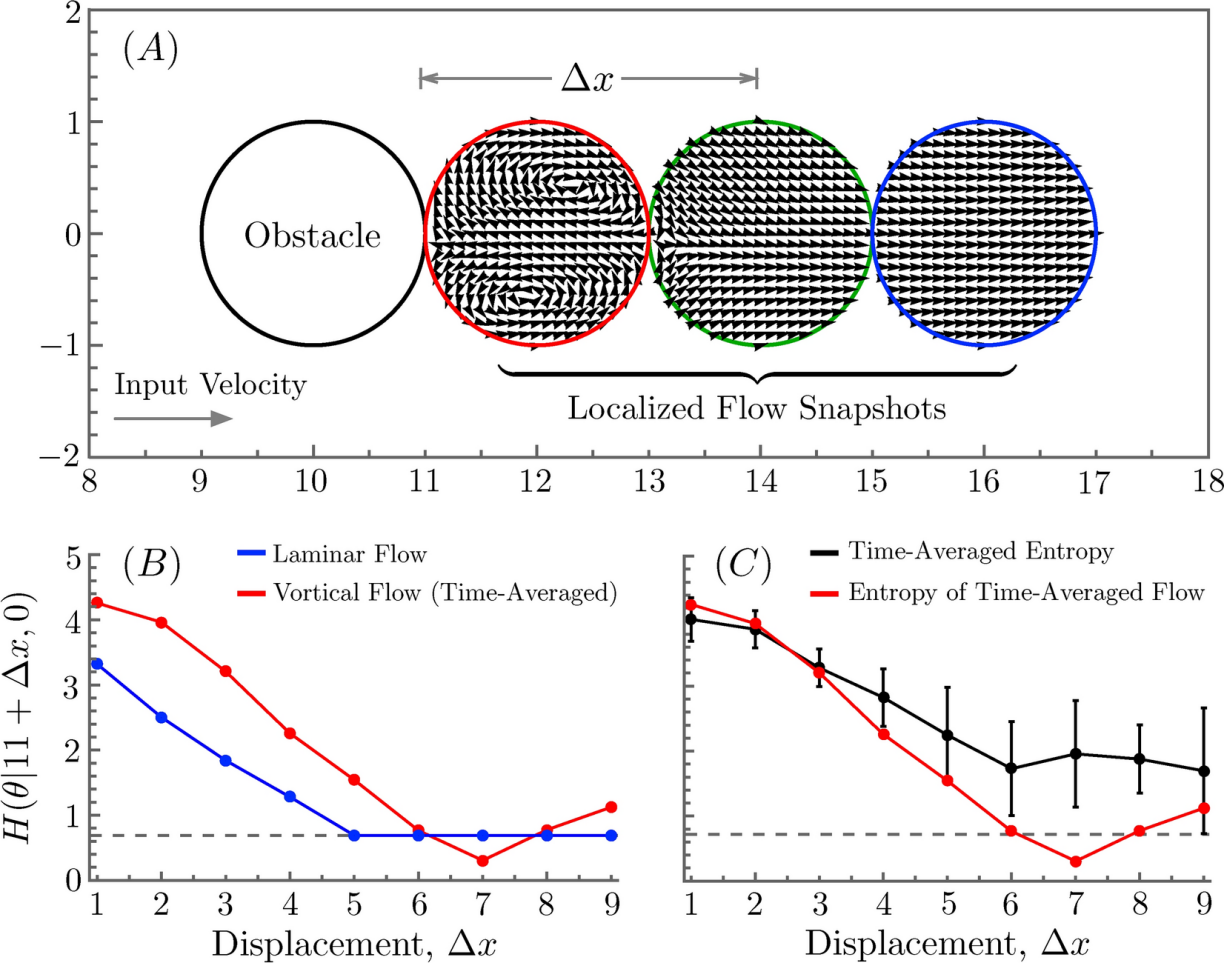
Local Orientational Vector Field Entropy (LOVE). (**A**) Channel flow around a circular obstacle. The inlet flow enters from the left of the channel with a uniform velocity parallel to the abscissa. To quantify how the obstacle’s impact on the flow diminishes with downstream distance, $\Delta x$, we compute the orientational entropy of the flow within a sequence of obstacle-shaped neighborhoods centered at successively further downstream locations. One can see from the plotted flow field snapshots (colored circles) that the induced vorticity immediately dowstream from the obstacle gradually dampens out, resulting in the flow field returning to its uniform inlet profile sufficiently far downstream. (**B**) The local orientational entropy of the flow field as a function of displacement from the downstream edge of the obstacle for laminar flow (blue) and time-averaged vortical flow (red). (**C**) The same entropy, but now comparing the time-averaged vortical flow (red) to the time-averaged entropy (black) over five different temporal snapshots of the dynamic flow field. For the latter curve, standard error bars are plotted alongside the data.

We start by selecting a sequence of subregions lying progressively further downstream from the obstacle, such that each subregion is equal in size and shape to the obstacle itself. Figure 5A illustrates this idea for a circular obstacle of unit radius. Next we compute the orientational entropy of the flow velocity field within each of these subregions using the standard Shannon entropy:

3 $$\begin{aligned} H(\theta |x_0,y_0)=-\sum _{i=0}^{2\pi /\Delta \theta }p(i\Delta \theta )\log \left( p(i\Delta \theta )\right) . \end{aligned}$$

In the above, $(x_0,y_0)$ denotes the central point of the subregion, the random variable $\theta$ is defined implicitly at each point (*x*, *y*) as $\tan \left( \theta (x,y)\right) \equiv v_y(x,y)/v_x(x,y)$, and $\Delta \theta$ is a discretization of the interval $[0,2\pi ]$ into $2\pi /\Delta \theta$ subintervals. The probability $p(\theta )$ is the geometric probability of finding points within the subregion centered at $(x_0,y_0)$ whose flow velocity vectors have orientation lying between $\theta$ and $\theta +\Delta \theta$. We will generally estimate this probability by taking a mesh of points within the subregion, evaluating the flow field at each point via CFD, and then histogramming the points based upon their flow vector orientation. By construction, this entropy will be zero when the flow field within the subregion is uniformly oriented, as it is by assumption at the channel inlet.

In Fig. 5B, we plot the entropy of Eq. (3) for the sequence of circular regions in Fig. 5A (plotted as a function of the distance *d* between each subregion’s center and the most downstream point of the obstacle) for two different flow velocities corresponding to laminar and vortical flow regimes. In the latter case, we use the time-averaged steady flow approximation discussed earlier in “Parameters of flow simulation” subsection. The lower dashed line on the curves marks the one-bit threshold ($\ln 2$ nats) below which the information is generally assumed to be indistinguishable from noise. The distance at which this threshold is first reached is how we will quantify the decorrelation length scale.

Note that the entropy of the laminar flow plateaus at the one-bit threshold because the flow field far from the obstacle consists of an upper-half plane of vectors with a very small downward component and a lower-half plane of vectors with a very small upward component. Because we always choose the first discretized, angular subinterval to begin at $\theta =0$, these two classes of vectors will always fall into different bins. Since there are, by the symmetry of the system geometry, an equal number of each, this leads to a Shannon entropy of one bit.

The entropy of the vortical flow case is more interesting, since we have used a time-average of the real dynamic flow field. To quantify how much information is lost by this approximation, we can compute the orientational entropy for different fixed time values of the true flow and average over those entropy values. This time-averaged entropy is plotted (with standard error bars) vs. the entropy of the time-averaged flow in Fig. 5C. Near to the obstacle, the time-averaging is a good approximation, but far from the obstacle, it underestimates the true complexity of the flow by a statistically significant extent. This suggests that the decorrelation length for high Reynolds number flows may be considerably larger than what one estimates from time-averaged CFD calculations.

It should be noted that even if the decorrelation lengths of each obstacle in a domain of interest can be estimated, optimal subdomain divisions that eliminate prediction error will often be unattainable. For example, if the downstream distance between two obstacles is less than the decorrelation length of the upstream obstacle, then it will be impossible to separate the obstacles into optimally chosen subdomains. At that point, one must either accept the prediction error that will arise from a suboptimal patching, or one must keep both obstacles in a single subdomain and train the deep-learning model on a broader range of possible subdomain obstacle configurations.

**Figure 6:**
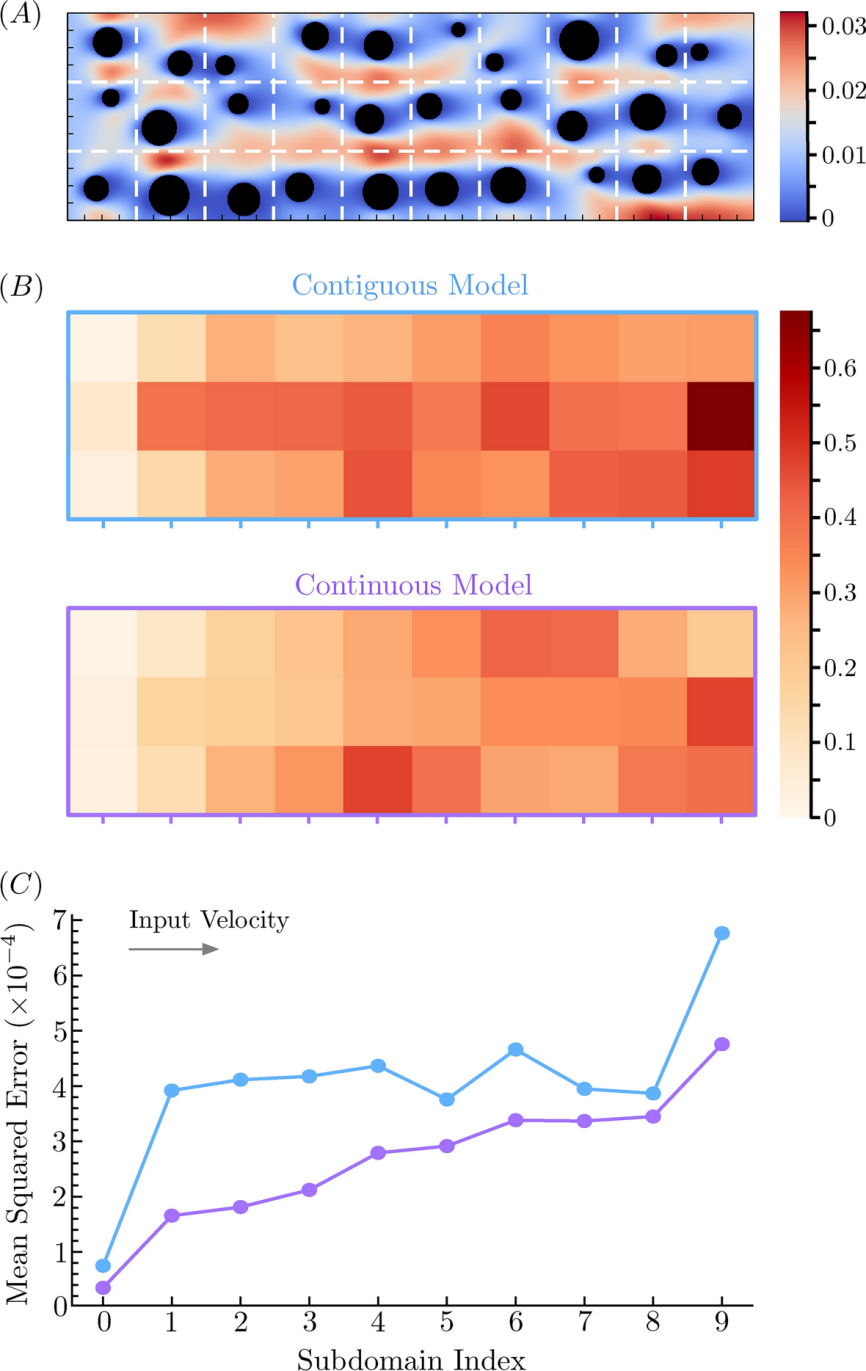
Error propagation in large domains. (**A**) Domain decomposition of a single $1280 \times 384$ domain into set of $128 \times 128$ subdomains. Error bars are scaled by a factor of $10^3$ for visual clarity. (**B**) Pixel level mean-square error (MSE) of each subdomain, evaluated on CFD data in a $1280 \times 384$ domain resolution. As expected, subdomain level errors compound as distance increases from the inlet of constant flow (here on the left hand vertical boundary). (**C**) Mean pixel-level MSE of the central row for each model, comparing Contiguous model predictions (blue) against Continuous model predictions (purple). Inlet direction is from left to right.

Aside from choosing the subdomain boundaries in a manner that best minimizes prediction error, there are three additional ways of decreasing this error: reduce the error of the Contiguous model, increase the performance of Continuous model, or a combination of both. We seek to assess the degree to which errors compound with increasing distance from the constant source flow, e.g., how MSE varies with *y* in Fig. 4. We trained the Contiguous model on a $128 \times 128$ pixel CFD dataset and the Continuous model on a $384 \times 384$ pixel CFD dataset. Finally, we used these models to predict velocity field components in domains of size $1280 \times 384$ and $1280 \times 640$ pixels. This provides an opportunity to explore how the Contiguous and Continuous models perform when predicting velocity fields for domains not represented by training data. Figure 6A shows how we decomposed a single $1280 \times 384$ domain into a set of $128 \times 128$ subdomains.

Figure 6B illustrates how these models perform. Although both the Contiguous and Continuous model predictions reveal an error that increases with *x*, MSE values tend to be lower for the Continuous model predictions. Quantitatively, the average MSE in the $1280 \times 384$ domain for the Contiguous model is 0.0033, whereas it is 0.0027 for the Continuous model, which represents an $18 \%$ reduction in the MSE. Errors are lowest near the inlet, and gradually increase towards the outlet. This is verified by Fig. 6C, which shows MSE for increasing *x* ($y=1$). This figure also illustrates that boundary effects may play a significant role in the behavior of velocity field predictions in this model. In practice, larger domains may be required to reduce the effect of “no slip” domain boundaries on centralized velocity field predictions.

## Discussion

While machine learning methods can be used for accurate flow prediction in complex environments, such as for urban structures^30^ or turbulent fields^31^, generalizing these approaches to domains of arbitrary size and complexity remains a challenging problem. One reason is that flows near and around obstacles depend on factors associated with the fluid (i.e., Reynolds number) or domain (i.e., boundary conditions), and fixing either of these conditions puts bounds on the validity of the estimated fields. Thus, if we seek broad applicability, then we should seek the fewest set of model restrictions that together provide the most accurate flow predictions. To this end, our approach has been to deconstruct certain types of domains into individual obstacles that each maintain some level of geometrical similarity, so that a single neural network model can be used to predict flows near all structural boundaries of the domain. Flows between these structural surfaces, at a scale on the order of the obstacle diameter, are predicted using a second neural network model in series with the first. Together, this serial-modeling approach allows for rapid prediction of flows in domains that can be represented by a disjoint set of structural elements. This type of domain is common, for example, in urban and periurban areas, wherein buildings conform to a common structural motif that affects ground-level velocity fields.

Another relevant length scale is the grid size used to digitize individual domains for read-in by the model. Thus, we investigated how flow patterns can be affected when this input resolution is varied. Although our choice of grid size is somewhat arbitrary, it is dense enough to capture variation in the relevant velocity fields near individual obstacles, but not so dense that producing a large enough cohort of CFD-generated training datasets becomes computationally intractable.

Our approach can also be trained to predict flows with a variable inlet velocity, which, in the case of urban wind flow prediction, permits model parameterization in terms of current meteorological conditions. In the specific case of aerial dispersion of chemicals throughout an urban environment, our predicted flows are considered as the advective field of a drift-diffusion model of molecular dispersion. This advection field plays a central role because concentration fluctuations decorrelate in relationship with the velocity fluctuations of the advection field, and spatial heterogeneity in the flow patterns is determined by the sequence of obstacles in the flow path. The popular Gaussian plume approach, which is common due to its mechanistically simple description and fast compute times, evolves concentrations according to unidirectional advection fields decoupled from any obstacles, and thus will only poorly predict dispersion across spatially heterogeneous domains at the distances considered here. To illustrate this point, consider that studies of hydrodynamic flow in porous media show that fluid velocities become decorrelated at moderate distances^32^, beyond which the flow can be approximated with Darcy’s law. Although one might not be able to perfectly map the structural elements of our model to the geometrical complexities of all urban locations, it can nevertheless be of value by adding short-distance spatial fidelity to flows ignored by the Gaussian plume approach.

Benchmarking reveals how the inclusion of different CNN architectures benefits the predictive accuracy of the model. Here we used a five-fold cross-validation approach to show that the introduction of gating, residual, and recurrent connections in the neural network architecture can benefit flow pattern prediction. When additional data are used for model training, its accuracy (reduction in the loss) increases in an exponential manner. For example, the scaling behavior of our Local Contiguous model, illustrated by the inset of Fig. 3, shows that a reduction in the loss by a factor of 10 requires nearly 35, 000 additional CFD simulated training samples. We were able to extend this Local Contiguous model to describe arbitrarily large domains with a serial domain-linking methodology, but noticed that error in the reconstructed domains is larger near edges and boundaries. To address this discrepancy, we applied a second deep learning model to these predictions to approximate smooth and continuous flows at these locations, termed the Continuous model. By construction, our method compounds the errors between adjacent domains. This is evident from Fig. 6, wherein the error increases monotonically with distance further into the domain and away from the velocity source. This means that although the Continuous model effectively reduces absolute error across the whole of the domain, it produces consistent and similar error between adjacent single-object domains. Thus, $\text {MSE}{\left( n \right) } = n \, \Delta \epsilon$, wherein $\Delta \epsilon$ is error accrued across adjacent domains, and *n* is the number of adjacent domains from the velocity source. For the the Contiguous model, this error appears domain dependent, i.e., ${\Delta \epsilon }{\left( n \right) }$. Indeed, Fig. 6 suggests that ${\Delta \epsilon }{\left( n \right) } \approx 0$ for $n=1-8$. In this case, model performance cannot be easily predicted when extrapolated to arbitrary scales, which is a problem solved by our Continuous model.

In summary, our analysis shows that reconstructing spatially-extensive velocity fields, as achieved by “stitching” individual predictions together, is a feasible strategy for predicting velocity fields that extend across larger domains constructed from spatially disjoint sets of obstacles, although a subsequent “smoothing” transformation of fields near the boundaries is still required to fulfill continuity requirements. We have shown that our models are reasonably predictive in domains larger than that of training data, at least to a reasonable level of error. Toward that end, we find that the error from our approach scales with the size of the domain, which, although not unexpected, emphasizes the point that fast, high-resolution fluid flow prediction remains an open problem. Both gated residual U-net and Recurrent Residual U-net architectures performed well for this flow prediction task. However, gated residual U-net is comparably lightweight and stable, with a faster training cycle. Due to a need for multiple feature channels, both our Contiguous and Continuous models can predict the flow for multiple inlet velocities and in the presence of topologically adjacent obstacles. Currently, our analysis focuses on two-dimensional flow around convex obstacles, but could be improved by an extension to three-dimensional domains. We can expect the predictive error in a three-dimensional model to grow faster than our current two-dimensional approach, simply because the volume of a cube will encode more velocity states than that of a planar patch. We have already seen that our model exhibits an increase in predictive error as fields become more complex. Thus, an extension of our approach to three-dimensions might require consideration of a more complex Contiguous model architecture (e.g., additional network layers). We suspect that enforcing additional physics-based constraints, such as with mass and energy, may reduce the training time of the architecture. We also intend to explore flow around nonconvex obstacles and higher Reynolds number where data-driven approaches have been able to identify scale invariant characteristics^33^. Ultimately, our approach is applicable to the velocity fields of heterogeneous domains that can be coarse-grained to a scale below the decorrelation length of local field density fluctuations. Not only is this relevant for urban wind velocity field prediction, but also for situations potentially as diverse as fluid flow in porous systems below a percolation threshold or pumped flow in dispersed granular media.

## Data Availability

Data is available upon request from the corresponding author.
